# Panel Sequencing of Primary Cutaneous B-Cell Lymphoma

**DOI:** 10.3390/cancers14215274

**Published:** 2022-10-27

**Authors:** Marion Wobser, Patrick Schummer, Silke Appenzeller, Hermann Kneitz, Sabine Roth, Matthias Goebeler, Eva Geissinger, Andreas Rosenwald, Katja Maurus

**Affiliations:** 1 Department of Dermatology, Venereology and Allergology, University Hospital Würzburg, 97080 Würzburg, Germany; 2 Comprehensive Cancer Center Mainfranken, University Hospital Würzburg, 97080 Würzburg, Germany; 3 Institute of Pathology, University of Würzburg, 97080 Würzburg, Germany; 4 Pathology Practice, 85049 Ingolstadt, Germany

**Keywords:** B-cell lymphoma, primary cutaneous follicular B-cell lymphoma, targeted sequencing

## Abstract

**Simple Summary:**

Primary cutaneous follicular B-cell lymphoma (PCFBCL) is a rare lymphoma subtype of the skin. Its biological behavior is mostly indolent, with slowly growing skin lesions exhibiting high response rates to topical or systemic therapy. Owing to the fact that there are only few studies addressing the underlying molecular basis of PCFBCL, the purpose of our study was to investigate the presence of oncogenic mutations in 10 cases of PCFBCL. By using hybridization-based panel sequencing of 40 lymphoma-associated genes, we identified genetic alterations within 15 of the selected target genes. Somatic mutations in *TNFRSF14, CREBBP, STAT6* and *TP53* genes were among the most commonly identified oncogenic alterations. The presence of any of these mutations was not associated with clinical features such as relapse or extent of skin lesions. To conclude, the identification of such genetic alterations helps to discriminate PCFBCL from cutaneous pseudo-lymphoma, and thus provide an additional diagnostic tool in difficult-to-diagnose cases.

**Abstract:**

Background: Primary cutaneous follicular B-cell lymphoma (PCFBCL) represents an indolent subtype of Non-Hodgkin’s lymphomas, being clinically characterized by slowly growing tumors of the skin and common cutaneous relapses, while only exhibiting a low propensity for systemic dissemination or fatal outcome. Up to now, only few studies have investigated underlying molecular alterations of PCFBCL with respect to somatic mutations. Objectives: Our aim was to gain deeper insight into the pathogenesis of PCFBCL and to delineate discriminatory molecular features of this lymphoma subtype. Methods: We performed hybridization-based panel sequencing of 40 lymphoma-associated genes of 10 cases of well-characterized PCFBCL. In addition, we included two further ambiguous cases of atypical B-cell-rich lymphoid infiltrate/B-cell lymphoma of the skin for which definite subtype attribution had not been possible by routine investigations. Results: In 10 out of 12 analyzed cases, we identified genetic alterations within 15 of the selected 40 target genes. The most frequently detected alterations in PCFBCL affected the *TNFRSF14*, *CREBBP*, *STAT6* and *TP53* genes. Our analysis unrevealed novel mutations of the *BCL2* gene in PCFBCL. All patients exhibited an indolent clinical course. Both the included arbitrary cases of atypical B-cell-rich cutaneous infiltrates showed somatic mutations within the *FAS* gene. As these mutations have previously been designated as subtype-specific recurrent alterations in primary cutaneous marginal zone lymphoma (PCMZL), we finally favored the diagnosis of PCMZL in these two cases based on these molecular findings. Conclusions: To conclude, our molecular data support that PCFBCL shows distinct somatic mutations which may aid to differentiate PCFBCL from pseudo-lymphoma as well as from other indolent and aggressive cutaneous B-cell lymphomas. While the detected genetic alterations of PCFBCL did not turn out to harbor any prognostic value in our cohort, our molecular data may add adjunctive discriminatory features for diagnostic purposes on a molecular level.

## 1. Introduction

Primary cutaneous follicular B-cell lymphomas (PCFBCL) belong to the indolent lymphoma subtypes of the skin [1]. Clinically, patients present with mostly solitary or few papules, plaques or tumors exhibiting slow growth dynamics [2]. Albeit cutaneous relapses may occur in about 20% of patients with PCFBCL, prognosis is nevertheless excellent and systemic dissemination to nodal or visceral sites is rare [3,4,5]. Hence, beyond a reasonable watch-and-wait strategy, therapeutic approaches mainly rely on skin-directed treatment [6,7,8].

Discrimination of PCFBCL from other cutaneous B-cell-rich inflammatory or neoplastic lymphoid infiltrates, i.e., B-cell pseudo-lymphoma, CD4+ T-cell lymphoproliferation, primary cutaneous diffuse large B-cell lymphoma (PCLBCL) and primary cutaneous marginal zone lymphoma (PCMZL), on histopathological grounds may be challenging [5,9]. Clinical [10] and histological [11,12,13] variants of PCFBCL such as clear-cell or spindle cell subtypes expand the wide spectrum of heterogeneous differential diagnoses. In addition, discrimination from secondary skin manifestation of systemic follicular B-cell lymphomas (FBCL) is of clinical relevance due to the divergent biological behavior of primary cutaneous versus systemic B-cell lymphomas.

Hence, final diagnosis relies on close clinicopathological correlation, immunohistochemical work-up as well as clonality analysis of the immunoglobulin heavy chain.

During the last years, a better insight into the chromosomal alterations, gene expression profiles and the mutational landscape of PCLBCL [14,15,16,17,18] and PCMZL [19,20] has provided deeper knowledge of the molecular pathogenesis of these cutaneous lymphoma subtypes, mirroring the respective biological behavior. In addition, these research activities delineated subtype-specific molecular markers which may serve as additional tools for better diagnosis [17,19] or risk stratification [21,22], and finally may pave the way for novel targeted treatment options based on the underlying molecular profile [23]. In contrast, until recently [24,25,26], the molecular pathogenesis of PCFBCL has remained widely elusive.

Therefore, we investigated the mutational profile of patients presenting with PCFBCL by targeted deep sequencing and correlated these molecular data with the clinical course of respective patients. 

## 2. Materials and Methods

### 2.1. Patients and Tissue

The study included 10 patients with PCFBCL. Approval of the entire study was obtained from the Ethics committee at the Medical Faculty of the University of Würzburg, Würzburg, Germany (ethics code 115/15).

Based on clinical, histological and immuno-phenotypical features, these indolent cutaneous B-cell lymphomas could clearly be attributed to the subtype of PCFBCL consistent with the current ISCL and WHO/EORTC recommendations on diagnosis and classification of cutaneous lymphomas [27,28].

Two additional ambiguous cases of atypical B-cell-rich infiltrates were included (cases #2 and #3). In these two cases—due to technical reasons/tissue quality—a final subtype attribution of PCFBCL versus PCMZL could not be drawn by certainty. 

Patients were diagnosed, staged, treated and monitored according to national and international guidelines [8,27,29] at the Department of Dermatology, University Hospital Würzburg, Germany. Systemic lymphoma or systemic involvement were excluded in each case. During the further disease course, no patient experienced systemic dissemination, and none died of her/his lymphoma. Patients’ characteristics are summarized in Table 1.

Histopathological examinations were based on HE- and Giemsa-stained slides as well as on immunohistochemistry using a standard panel of antibodies. Microscopic evaluation of stained slides was performed independently by three (dermato-)pathologists of the Department of Dermatology, University Hospital Würzburg, and the Institute of Pathology, University of Würzburg. PCR clonality analyses of the *FR2A* and *FR3A* regions of the immunoglobulin heavy chain (according to the Biomed-2 protocol) were performed in 7 of 10 cases of PCFBCL and revealed a clonal B-cell population in 2 cases (cases #5 and #10), while the remaining cases showed a polyclonal infiltrate [30]. BCL2 expression in B-cells was present in 5/10 cases, and 1 of these cases showed a *BCL2*-rearrangement by FISH analysis (patient #9) (Table 1). Fresh-frozen cryopreserved tissue was available from all patients for molecular analysis. Blood samples were obtained from patients #1–7 as matched control samples. An overview on key features of primary cutaneous B-cell lymphomas is provided in Table 2.

### 2.2. Targeted Sequencing

Motivated by our recent success to delineate recurrent somatic mutations in PCMZL, we took advantage of a similar methodological approach by sequencing a panel of 40 cardinal genes being crucially implicated into the pathogenesis of lymphoid neoplasms [20]. The gene list can be found in Supplementary Table S3. 

### 2.3. DNA Extraction

Genomic DNA from tissue specimen and corresponding blood samples were extract-ed with the DNeasy Blood and Tissue Kit (Qiagen, Hilden, Germany). DNA quantitation was assessed by the Qubit dsDNA Broad-Range Assay (Life Technologies, Darmstadt, Germany).

### 2.4. Hybridization-Based Panel Sequencing

The HaloPlexHS Target Enrichment System (Agilent Technologies Inc., Santa Clara, CA, USA) including 40 full-length coding lymphoma-associated genes (Supplementary Table S3) was used for library preparation, strictly according to the manufacturer’s protocol. The captured libraries were amplified during 23 PCR cycles. The libraries were sequenced on the MiSeq platform with a 150 bp paired-end sequencing approach (Illumina, San Diego, CA, USA).

### 2.5. Sanger Sequencing

Sequencing analysis of the *FAS* target region was performed as described previously [20].

### 2.6. FISH Analysis

Chromosomal rearrangements of the *BCL2* gene locus were analyzed using the ZytoLight SPEC BCL2 Dual-Color Break-Apart Probe (Zytovision, Bremerhaven, Germany) on formalin-fixed paraffin-embedded (FFPE) biopsy specimens. FBCL displaying break-apart signals in ≥7% of cells were considered to harbor a rearrangement.

### 2.7. Bioinformatical Data Analysis

Data analysis quality trimming: An initial quality assessment was performed using FastQC, version 0.11.3 (http://www.bioinformatics.babraham.ac.uk/projects/fastqc/). Adapters and low-quality reads were trimmed from 151 bp paired-end reads using TrimGalore, version 0.6.1 (http://www.bioinformatics.babraham.ac.uk/projects/trim_galore/), powered by Cutadapt, version 2.3 (https://cutadapt.readthedocs.io/en/stable/).

Read alignment: The trimmed reads were mapped to the human reference genome (hg19) using BWA MEM, version 0.7.17 [31], and sorted and indexed using Picard, version 1.125 (available online at http://broadinstitute.github.io/picard/), and SAMtools, version 1.3 [32], using htslib, version 1.3. Local insertion‒deletion realignment was executed with GATK, version 3.5 [33].

According to the manufacturer’s instructions, a deduplication step was added using the AgilentMBCDedup tool, version 1.0, provided by Agilent (Santa Clara, CA, USA). GATK, version 3.5, was also used for coverage calculations.

Somatic variant calling: MuTect1, version 1.1. [34], VarScan2, version 2.4.1 [35], Scalpel, version 0.5.3 [36] and MuTect2 (that is integrated in the GATK4, package, version 4.0.11.0 [33]), were used to identify somatic single nucleotide variants and small somatic insertions or deletions (Supplementary Table S2). All variants were annotated with ANNOVAR, version 2019-10-24 [37]. Variants were considered somatic if they have an impact on the protein sequence or if they affect a splice site, if they are rare in the population (below a frequency of 2% in 1000g2015aug_all, ExAC_nontcga_ALL, gnomAD_exome_ALL and gnomAD_genome_ALL), if the position is covered by at least 20 reads and the alternative allele is covered by at least 5 reads and if they comprised at least 2% and are absent in the matched normal blood sample. 

All variants were visually examined using the Integrative Genomics Viewer, version 2.3.68, to check for their validity [38]. All detected variants are presented in Supplementary Table S1, including their biological relevance according to Clinvar and cbioportal databases [39,40].

## 3. Results

### 3.1. Recurrent Somatic Mutations Are Present in PCFBCL

**Figure 1:**
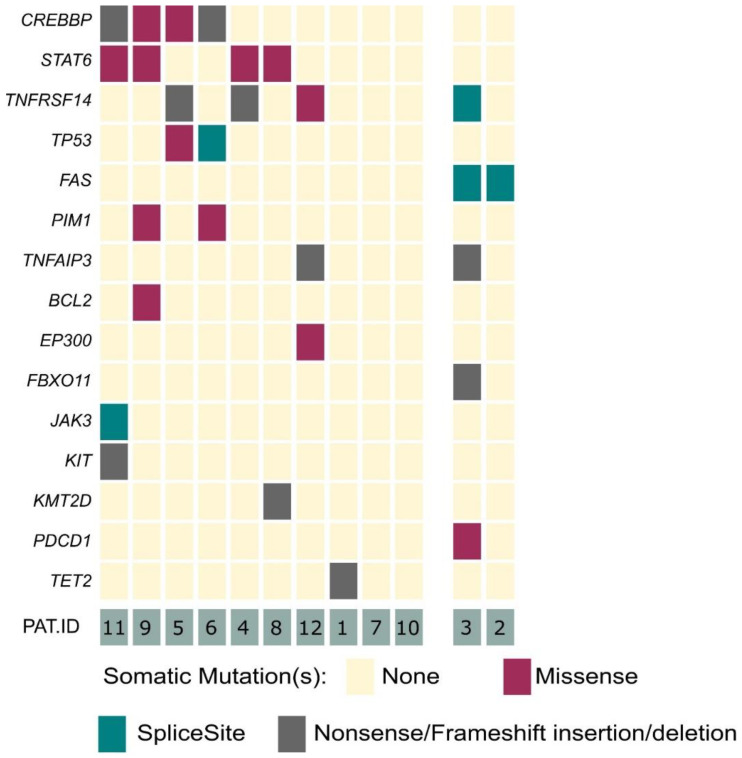
Overview of mutations in primary cutaneous FBCL. Graphical illustration of detected variants within 15 of the analyzed 40 lymphoma-associated genes. Left side of graph: ten cases of PCFBCL (from top to bottom, most frequently to less frequently mutated genes). Right side of graph: two cases of arbitrary B-cell-rich cutaneous infiltrates being finally reclassified as PCMZL.

Our panel included 40 genes, which are associated with B- and T-cell development, as well as lymphomagenesis [20]. In 10 out of 12 analyzed cases, we identified genetic alterations within 15 of the selected 40 target genes (Figure 1). Within the clear-cut PCFBCL cases (*n* = 10), the most frequently detected alterations affected the *CREBBP* (40%), *STAT6* (40%) and *TNFRSF14* (30%) genes. Moreover, *TP53* and *PIM1* were each affected in two patients (20%). In single cases, mutations in seven further genes, affecting different cellular processes, were detected (*TNFAIP3*, *BCL2*, *EP300*, *JAK3, KIT*, *KMT2D* and *TET2*).

In addition to these already known oncogenic alterations, we identified novel, presumably pathogenically relevant mutations in our 10 cases of PCFBCL. These mutations mainly affected genes with similar biological functions as the mutated genes mentioned above. We identified alterations in genes inflicting the JAK/STAT pathway (*JAK3* mutation) or orchestrating epigenetic modification and cell metabolism (*TET2*).

For the first time, we identified a *BCL2* gene mutation in a case of PCFBCL. *BCL2* mutations have until now only been detected in systemic FBCL counterparts. One of the five BCL2-positive PCFBCL cases, as determined by immunohistochemical staining, harbored a *BCL2*-rearrangement by FISH analysis (patient #9). 

No alterations of the *FAS* gene were detected among these 10 clear-cut cases of PCFBCL.

### 3.2. Alterations of the FAS Gene May Serve as An Adjunctive Molecular Tool for Subtype Classification of Indolent B-Cell Lymphomas of the Skin

**Figure 2:**
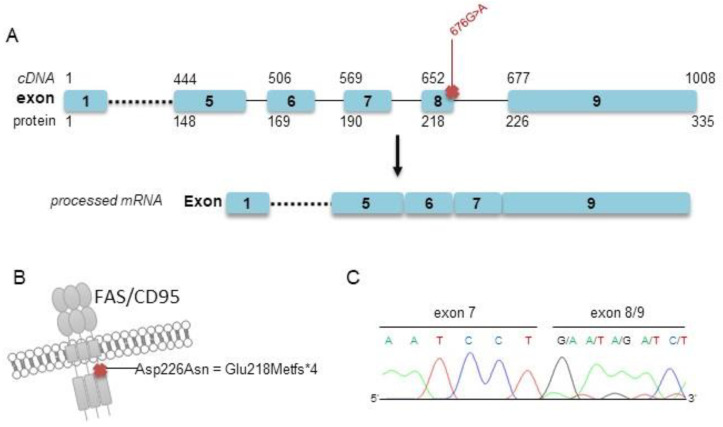
Localization of the detected splice site mutations within the *FAS* gene and confirmatory Sanger sequencing (patient #2). (**A**) Schematic illustration of the detected splice site mutation c.676G > A and processed mRNA lacking exon 8 of case #2. (**B**) Localization of the annotated mutation p.Asp226Asn at the protein level, leading to a truncated protein p.Glu218Metfs*4. (**C**) Results of Sanger sequencing of cDNA exhibiting a truncated *FAS* mRNA transcript lacking exon 8.

Our targeted sequencing analysis included two further arbitrary cases (cases #2 and #3) for which a decisive classification as either PCFBCL or PCMZL could not be made based on clinical and histopathological grounds mainly due to small biopsies and limited material. Both samples were polyclonal in PCR analysis of immunoglobulin heavy-chain genes. Both cases showed somatic mutations within the *FAS* gene, as evidenced by targeted sequencing. As comparable mutations of the *FAS* gene have previously been designated by us and others as subtype-specific recurrent alterations in PCMZL [19,20], we finally favored to classify these two ambiguous cases as PCMZL rather than PCFBCL based on these molecular findings. Of note, one of these cases also harbored one nonsynonymous, exonic, and one splice site mutation affecting the *TNFRSF14* gene, as well as a deleterious *TNFAIP3* mutation. Representative *FAS* Sanger sequencing from cDNA of patient #2 confirmed the splice site defect already detected by targeted sequencing of genomic DNA (Figure 2).

## 4. Discussion

Here, we described a cohort of PCFBCL patients in whom we detected recurrent mutations in the *TNFRSF14*, *STAT6* and *CREBBP* genes. These are involved in diverse signaling pathways that regulate immunological processes and drive tumorigenesis [41]. Such mutations have already been detected in PCFBCL in previous reports, albeit with variable frequencies putatively due to different methodological approaches and small case series. In the cohort of PCFBCL patients (*n* = 22) reported by Barasch et al., the frequencies of mutations were found to be 40% for *TNFRSF14*, 25% for *CREBBP*, 25% for *TNFAIP3* and 17% for *STAT6*, respectively, as evidenced by targeted sequencing [25]. This is slightly different from our findings revealing *TNFRSF14* mutations in only 20% of the analyzed cases, being more close to the data published by Gango et al., who used Sanger sequencing [24], and Zhou et al., who applied exome sequencing [26]. However, one has to keep in mind that, beyond the different methodological approaches applied in these studies, especially small patient numbers limit the data interpretation of previous investigations, including our own study.

Functional loss of TNFRSF14 is implicated in orchestrating the composition of the immune environment via the ligation of the B- and T-lymphocyte attenuator (BTLA) and regulating the expression of stroma-derived cytokines [42]. These genetic events may thus contribute to the dense infiltration of tumor-associated immune cells, as observed histologically in PCFBCL.

While expression of CD10 and BCL2 are hallmarks of systemic FBCL, these markers are rather inconsistently present in PCFBCL, implying a more variable immunophenotype [43,44]. Likewise, at the gene level, the t(14;18) translocation involving the *BCL2* gene is commonly detected in systemic FBCL, whereas in PCFBCL it remains restricted to rare cases [45]. In this respect, while 5/12 samples of our series showed positivity of BCL2 by immunohistochemistry, only one of our cases showed the pathognomonic *BCL2* translocation and, in addition, two biologically uncharacterized point mutations within the *BCL2* gene. The latter represents a novel finding with respect to PCFBCL. Of note, none of these cases showed any evidence of systemic involvement at primary diagnosis or during the further disease course, as assessed by meticulous staging examinations and close follow-up monitoring. Hence, alterations of the *BCL2* gene, either by translocation or mutation, do not seem to convey any biological significance and prognostic issue in PCFBCL. Altogether, irrespective of the presence of somatic mutations, all patients with PCFBCL in our series exhibited an indolent clinical course, and the molecular profile did not impact tumor stage or the frequency of cutaneous relapses.

Therefore, although investigated in a rather small patient cohort, our data provide further evidence that albeit PCFBCL differ from their systemic counterparts with respect to biological behavior, both entities nevertheless share similar alterations (rearrangements, mutations) in a limited set of key genes, however with notable variable frequencies according to hitherto available data [26,46]. Along with *EZH2* mutations, genetic alterations of the chromatin-modifying genes *KMT2D* and *CREBBP* are considered as hallmarks of systemic FBCL [47]. A recent literature review and meta-analysis compiling more than 1000 cases of systemic FBCL has provided frequencies of 72% for *KMT2D*, 65% for *CREBBP*, 47% for *BCL2* and 33% for *TNFRSF14* [25]. The most prevalent oncogenic aberrations in our case series of PCFBCL were indeed recurrent mutations in *CREBBP* (40%), while alterations of *KMT2D* were only rarely detected. CREBBP is a highly conserved lysine acetyltransferase serving as a transcriptional coactivator, regulating cell growth and division. Loss-of-function mutations of *CREBBP* are implicated as early events in the pathogenesis of systemic FBCL, being present in more than 70% of cases [48]. To conclude, systemic FBCL and PCFBCL share an overlapping mutational profile, however with different frequencies. In contrast to systemic FBCL, the occurrence of translocations of *BCL2* is not a common feature of PCFBCL. This may be a relevant molecular finding to explain the distinct biological behavior, bearing in mind that PCFBCL displays a low propensity for systemic dissemination and an almost unrestricted overall survival, which was again evidenced in our patient cohort. This overlapping mutational profile, however, hampers further differentiation of PCFBCL from secondary skin infiltrates of nodal FBCL based on molecular investigations. Hence, compiling clinical findings and histology in conjunction with appropriate staging examinations remains the gold standard for final diagnosis and subsequent subtype-specific treatment decisions.

Despite the availability of a wide range of diagnostic antibodies for tissue staining and established morphological and immuno-phenotypical criteria for pathological diagnosis of cutaneous B-cell lymphomas, secure diagnosis of PCFBCL may still be difficult in selected cases. As already evidenced with the example of PCMZL [20], the detection of somatic mutations in PCFBCL may nevertheless help to differentiate this indolent lymphoma subtype from reactive, B-cell-rich infiltrates, such as Borrelia-associated B-cell pseudo-lymphoma or CD4^+^ T-cell lymphoproliferation, and last but not least, from other cutaneous B-cell lymphomas such as PCLBCL or PCMZL.

Especially differentiation of PCFBCL from PCLBCL may be a challenge when a high proportion of large B-cells are present in PCFBCL [17,49]. However, in that specific scenario of large B-cell lymphoma, an accurate lymphoma subtype attribution is crucial owing to the fact that PCLBCL runs an adverse clinical course and demands more extensive staging examinations as well as aggressive upfront treatment. Beyond different chromosomal changes [18] and gene expression profiles [14], none of the recurrent and pathognomonic genetic aberrations of PCLBCL—such as oncogenic *MYD88* or *CD79B* mutations or *TNFAIP3* deletions [15,16,17]—were present in any of our analyzed cases of PCFBCL and, vice versa, the herein detected somatic mutations in our PCFBCL cohort are not a common feature of PCLBCL. Hence, our findings are in line with previous data that clearly separate these lymphoma subtypes, by means of molecular profiling, as belonging to biologically distinct entities [15,16,50].

Targeted sequencing is cost- and time-intensive and not readily available for the pathologist within the daily routine. Beyond deep sequencing approaches, the method of Sanger sequencing is simple, fast and may be widely used. Splice site mutations of the *FAS* gene affecting its functionally relevant death domain are rather specific for PCMZL, being detected in >60% of investigated cases and only rarely in other subtypes of marginal zone lymphoma [19,20]. Similar alterations of the *FAS* gene have not been detected in FBCL according to hitherto published data [24,25,26]. In our cohort, we detected respective genetic alterations in two ambiguous cases of atypical B-cell-rich cutaneous infiltrates. Consecutively, owing to the fact that such *FAS* alterations have only been detected previously in clear-cut cases of PCMZL, we favored the diagnosis of PCMZL in these two grey-zone cases. As the identification of these *FAS* alterations is amenable to Sanger sequencing, this finding may further be exploited for diagnostic issues in the future. Nevertheless, one must keep in mind that both lymphoma subtypes mostly follow an indolent clinical course so that there will be no impact on therapeutic decisions or prognostic outcome. 

The same applies for CD4+ T-cell lymphoproliferation, an indolent provisory cutaneous lymphoma entity [1], which may represent a diagnostic pitfall to misdiagnose as PCFBCL due to the high amounts of B-cells within the infiltrate. Genetic alterations have only been described in extraordinarily rare cases in this benign lymphoid proliferation [51]. The recently detected *DNMT3A* mutation being reported by the French lymphoma study group in one single case of their series of CD4+ lymphoproliferation has hitherto not been described in PCFBCL, and thus might represent a discriminatory molecular feature if validated in further studies.

To conclude, our deep sequencing data revealed recurrent mutations in PCFBCL, such as in *TNFRSF14*, *CREBBP* and *STAT6*, thus classifying this lymphoma subtype as a distinct lymphoma entity within the spectrum of cutaneous lymphomas based on molecular grounds. Beyond already known genetic alterations, we detected novel mutations in genes such as *BCL2* which impact cardinal mechanisms of tumorigenesis. Overlapping molecular alterations and immuno-phenotypical features in systemic FBCL and PCFBCL imply common mechanisms of pathogenesis. Nevertheless, the presence of somatic mutations in PCFBCL did not convey any prognostic issues in our patient cohort. Larger patient cohorts in multi-institutional approaches are warranted to confirm hitherto identified molecular aberrations in PCFBCL.

## Figures and Tables

**Table 1:** Patient characteristics. Tabular overview on clinical data of the ten included PCFBCL patients and two further arbitrary cases (cases #2 and #3)—being finally reclassified as PCMZL—with immunohistochemical and molecular findings of respective analyzed tissue specimen.

Pat ID	Sex	Age at Primary Diagnosis [Years]	Tumor Stage at Primary Diagnosis	Tumor Stage at Date Last Seen	Cutaneous Relapses	Number of Cutaneous Relapses	Systemic Dissemination	Treatment	Overall Survival Since Primary Diagnosis [Months]	Final Status	Clonality	bcl-2 Expression Immunohistochemistry	bcl2 FISH Analysis
1	male	39	T2 N0 M0	T2 N0 M0	yes	1	no	rituximab	117	alive	polyclonal	negative	negative
2	male	60	T2c N0 M0	T2c N0 M0	no		no	rituximab	75	dead	polyclonal	positive	negative
3	female	70	T2 N0 M0	T2 N0 M0	no		no	rituximab	131	alive	oligoclonal/polyclonal	positive	negative
4	male	54	T1 N0 M0	T2 N0 M0	yes	3	no	excision, radiation	200	alive	oligoclonal/polyclonal	negative	negative
5	female	70	T2 N0 M0	T2b N0 M0	yes	>5	no	excision, radiation, topical steroids, rituximab	117	alive	monoclonal	positive	negative
6	female	66	T1 N0 M0	T1 N0 M0	no		no	watch-and-wait	66	alive	not done	negative	negative
7	female	89	T1b N0 M0	T1b N0 M0	no		no	radiation	47	alive	not done	negative	negative
8	male	45	T1a N0 M0	T1a N0 M0	no		no	excision	23	alive	polyclonal	positive	negative
9	male	50	T1a N0 M0	T1a N0 M0	no		no	radiation	21	alive	not done	positive	positive
10	male	50	T1 Nx Mx	T1 Nx Mx	no		no	watch-and-wait	57	alive	monoclonal	negative	negative
11	female	53	T2 N0 M0	T2 N0 M0	yes	2	no	radiation, topical steroids	35	alive	not done	negative	negative
12	male	43	T2 N0 M0	T2 N0 M0	no		no	rituximab	107	alive	not done	negative	negative

**Table 2:** Characteristic clinical, histological and immuno-phenotypical findings of the three major subtypes of primary cutaneous B-cell lymphomas.

Lymphoma Subtype	Characteristic Clinical Findings	Histology	Common Immunphenotype	Common Treatment Modalities	Prognosis
Primary cutaneous follicular B cell lymphoma (PCFBCL)	slowly growing erythematous plaques, papules, tumors; often at the head	Diffuse or follicular dermal B-cell infiltrates, mostly centrocytes	CD20+, bcl2 - (+), bcl-6 +, MUM-1 -, CD21 + remnants of follicular dendritic cells networks	watch and wait, excision, radiation, rituximab	Favorable. 5-year survival rate: >95%
Primary cutaneous marginal zone lymphoma (PCMZL)	slowly growing erythematous plaques, papules, tumors; often at trunk and extremities	Diffuse dermal B-cell infiltrates with germinal centers and plasma cells	CD20+, bcl2 +, bcl-6 -, MUM-1 -, mostly IgG4 +	watch and wait, excision, radiation, rituximab	Favorable. 5-year survival rate: >95%
Primary cutaneous diffuse large B-cell lymphoma (PCLBCL)	rapidly growing ulcerated tumors; mostly at the legs	Diffuse dermal sheet-like B-cell infiltrates, mostly centroblasts, immunoblasts	CD20+, bcl2 +, bcl-6 -/+, MUM-1 +, IgM +, CD21 lack of follicular dendritic cells networks	rituximab-CHOP +/- radiation	Poor. 5-year survival rate: 20–60%

## Data Availability

Data will be provided upon request for reasonable academic studies by the corresponding author.

## Supplementary Materials

The following supporting information can be downloaded at: https://www.mdpi.com/article/10.3390/cancers14215274/s1. Supplementary Table S1: Somatic variants detected by hybridization-based panel sequencing and biological impact. Supplementary Table S2: Read statistics of hybridization-based panel sequencing. Supplementary Table S3: Gene list for hybridization-based panel sequencing.
